# PSMA2 promotes chemo- and radioresistance of oral squamous cell carcinoma by modulating mitophagy pathway

**DOI:** 10.1038/s41420-025-02286-2

**Published:** 2025-01-10

**Authors:** Chun-I Wang, Cheng-Yi Chen, Ting-Wen Chen, Chun-Chia Cheng, Shu-Wen Hong, Tsung-You Tsai, Kai-Ping Chang

**Affiliations:** 1 Department of Biochemistry, School of Medicine, China Medical University, Taichung, Taiwan; 2 Department of Cell Biology and Anatomy, College of Medicine, National Cheng Kung University, Tainan, Taiwan; 3 Institute of Bioinformatics and Systems Biology, National Yang Ming Chiao Tung University, Hsinchu, Taiwan; 4 Radiation Biology Research Center, Institute for Radiological Research, Chang Gung University/ Linkou Chang Gung Memorial Hospital, Taoyuan, Taiwan; 5 Department of Otolaryngology-Head & Neck Surgery, Linkou Chang Gung Memorial Hospital, Taoyuan, Taiwan; 6 College of Medicine, Chang Gung University, Taoyuan, Taiwan; 7 Molecular Medicine Research Center, Chang Gung University, Taoyuan, Taiwan

**Keywords:** Tumour biomarkers, Oncogenes

## Abstract

Oral cavity squamous cell carcinoma (OSCC) represents the most prevalent malignancy among head and neck squamous cell carcinomas (HNSCCs). Standard treatment modalities include surgical resection combined with radiation and chemotherapy. However, locoregional failure remains a critical issue affecting the prognosis of OSCC patients, largely due to tumor resistance against radiation or chemotherapy. In this study, we established a gene database related to OSCC recurrence and identified PSMA2 as a novel molecule influencing prognosis in OSCC patients. An independent Taiwanese cohort confirmed that elevated PSMA2 transcript levels were associated with poorer prognosis and contributed to the chemo- and radioresistance phenotype in OSCC. Furthermore, we confirmed that PSMA2 regulates cell cycle, mitochondrial dysfunction, and mitophagy, thereby contributing to carcinogenesis and resistance. Notably, mitophagy inducer exhibit antitumor effects in PSMA2-overexpressing OSCC xenograft mouse model. Collectively, our results provide a mechanistic understanding of the atypical function of PSMA2 in promoting OSCC recurrence.

## Introduction

OSCC represents the most prevalent aggressive form of head and neck squamous cell carcinomas (HNSCCs), with a discouraging 5-year survival rate of ~50% over the past few decades [1]. Surgical resection is the primary treatment approach for early-stage OSCC patients, while postoperative radiotherapy is typically recommended for those with advanced disease or adverse pathologic/treatment features. Concurrent chemoradiotherapy (CCRT) is employed for patients with high-risk features [2, 3]. However, the high rates of regional failure and distant tumor recurrence in OSCC remain significant challenges. More than 25% of OSCC patients experience locoregional recurrence or metastasis, often occurring within the initial 2 years of follow-up after OSCC treatment [4–6]. Despite recent advances in treatment protocols, the long-term survival rate of OSCC patients has stagnated at around 50% for the past two decades. This unsatisfactory outcome can be attributed to the frequent locoregional recurrence and heightened likelihood of distant metastasis driven by chemo- and radioresistance [7]. The persistently limited success in OSCC treatment underscores the urgent need for continuous efforts to enhance treatment modalities. To advance our understanding and contribute to personalized treatment and management of OSCC, there is a critical need for comprehensive and systematic analyses aimed at identifying specific markers for recurrence and prognosis in OSCC in the future.

The ubiquitin-proteasome pathway is widely distributed in eukaryotic cells and functions in the ATP/ubiquitin-dependent degradation of peptides [8]. Obsolete or damaged endogenous proteins are initially targeted for proteolysis through the attachment of a polyubiquitin chain, followed by rapid degradation to small peptides by the proteasome, with subsequent release and recycling of ubiquitin [9]. The 26S proteasome complex consists of two subcomplexes, namely the 19S regulatory complex and the 20S core complex. In addition to the canonical proteasome degradation pathway, emerging evidence suggests that degradation can also occur solely through the 20S proteasome, independent of the regulatory function of the 19S particle. Moreover, several biological pathways regulated by the uncapped 20S proteasome have been demonstrated, including proteolysis of intrinsically disordered proteins, oxidative stress adaptation, neuronal communication, and post-translational processes. Importantly, recent findings have indicated extracellular roles of the 20S proteasome, both as freely circulating particles in the plasma and encapsulated in extracellular vesicles [10]. Proteasome 20S Subunit Alpha 2 (PSMA2), one of the seven alpha subunits in the 20S proteasome complex, functions as a substrate entrance gate, participating in the degradation or recycling of defective proteins and proteolytic modification of numerous cellular regulatory proteins [11, 12]. Aberrant levels of PSMA2 have also been observed in breast cancer, colorectal cancer, and glioma. Elevated expression of PSMA2 has been correlated with poor survival in breast cancer patients [13] and it enhances proliferation, migration, and invasion in colorectal cancer and glioma [14, 15] while also being involved in immune and cellular stress responses in human lung cancer, ovarian cancer, leukemia and colorectal cancer [14, 16–18].

At present, it has been discovered that PSMA2 participates in both canonical and non-canonical proteasome degradation pathways. However, the clinical and pathological implications of PSMA2 in the tumorigenesis and recurrence of OSCC remain poorly understood. In the current study, we conducted a comprehensive analysis to identify novel recurrence-associated genes in OSCC and found that PSMA2 is an independent prognostic biomarker. We demonstrated its involvement in regulating chemo- and radioresistance phenotypes via the mitophagy pathway. Therefore, PSMA2 could be a useful prognostic marker and therapeutic target for OSCC.

## Results

### PSMA2 is significantly upregulated in OSCC and shows a strong correlation with OSCC recurrence

**Fig. 1:**
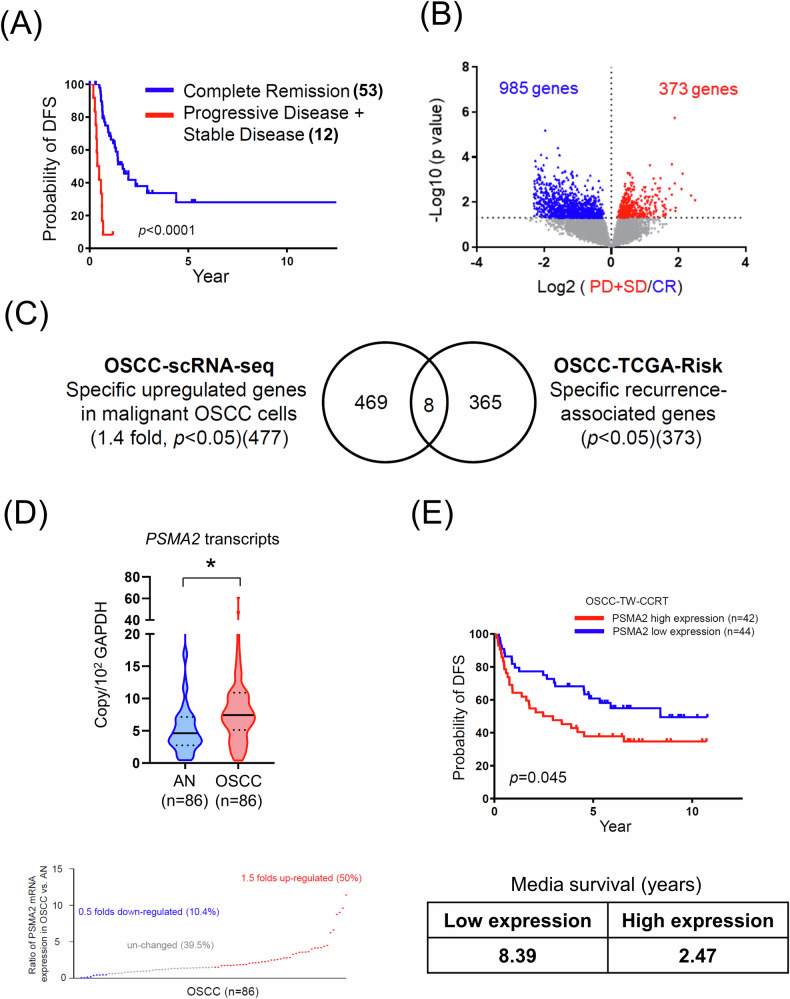
High PSMA2 expression predicts a poor response in the OSCC patients with CCRT in the TCGA and Taiwanese cohorts. **A** Kaplan–Meier survival curves showing the DFS rates for 53 patients with CR versus 12 patients with PD/SD. **B** Volcano plot of differentially expressed genes between CR and PD/SD. **C** Schematic diagrams show the comparison of OSCC-scRNA-seq with OSCC-TCGA-Risk. **D** PSMA2 transcript levels in 86 paired Taiwanese OSCC tissues were determined by qPCR. **E** Kaplan–Meier survival curves showing the DFS rates for patient subgroups stratified by high versus low PSMA2 expression (*p* = 0.0477, log-rank test).

To identify recurrence-associated genes in OSCC, we performed a comprehensive analysis of DEGs in 65 OSCC patients from TCGA database. Among these patients, 53 exhibited a complete remission (CR group) while 12 showed progressive disease or stable disease (PD/SD group). Patients in the PD/SD group displayed poorer disease-free survival (DFS) rates compared to those in the CR group, as demonstrated by Kaplan–Meier survival plot (Fig. 1A). Through further analysis, we identified 373 significantly upregulated genes (OSCC-TCGA-Risk) and 985 downregulated genes in the PD/SD group compared to the CR group (Fig. 1B). To identify novel recurrence-associated genes which are upregulated in tumor and correlated with poorer survival in OSCC, we compared OSCC-TCGA-Risk with OSCC-scRNA-seq, which containing 477 upregulated genes in malignant OSCC cells compared with non-malignant epithelial cells (unpublished data) (Fig. 1C). By this comparison, we found eight potential candidate genes are upregulated in malignant OSCC cells and associated with OSCC recurrence. Among these potential candidate genes, five have been previously reported as dysregulated proteins or genes in HNSCC, two have been reported as involved in HNSCC recurrence via literature search (Table 1). Among these three novel candidates, PSMA2 was selected for further study due to its significantly higher expression in malignant cells. Next, we used a validation-testing Taiwanese cohort, including 86 OSCC tissue specimens from tumors and corresponding adjacent normal tissue specimens to assess the prognostic value of PSMA2. All patients were primarily managed by surgical resection and subsequently underwent CCRT. Figure 1D showed consistently high expression of PSMA2 in OSCC tissues compared with the corresponding adjacent normal tissues. Importantly, patients with high PSMA2 expression exhibited poorer DFS rates than those with low PSMA2 expression (Fig. 1E). Collectively, these results indicated that PSMA2 is elevated in OSCC and higher expression is associated with a poorer prognosis, supporting a potential role of PSMA2 in cancer recurrence.

**Table 1:** List of 8 specific differentially expressed recurrence-associated genes in OSCC.

Gene name	M-T/NM-N^a^ in OSCC-scRNA-seq	*p*-value	Dysregulation in HNSCC (ref.)	SD + PD/CR^b^ in OSCC-TCGA-Risk	*p*-value	Recurrence-associated function in HNSCC (ref.)
SOD2	1.73	1.23E-04	Yes [23]	1.68	8.42E-03	Yes [28]
CYCS	1.61	3.18E-21	Yes [24]	1.48	6.51E-04	
LGALS7B	1.53	1.73E-14	Yes [25]	2.10	4.71E-02	Yes [29]
PSMA2	1.49	5.03E-11		1.34	2.02E-02	
LSM5	1.48	2.86E-05		1.35	4.86E-02	
PPIA	1.44	5.11E-46	Yes [26]	1.42	2.14E-02	
HIST1H2AC	1.42	7.08E-03		1.56	1.37E-02	
PCMT1	1.41	7.76E-12	Yes [27]	1.24	3.21E-02	

^a^The ratio of malignant cells in tumor tissues/non-malignant epithelial cells cells in adjacent normal tissues.

^b^The ratio of progressive disease or stable disease/complete remission.

### PSMA2 contributes to chemo- and radioresistance phenotype in OSCC cells

**Fig. 2:**
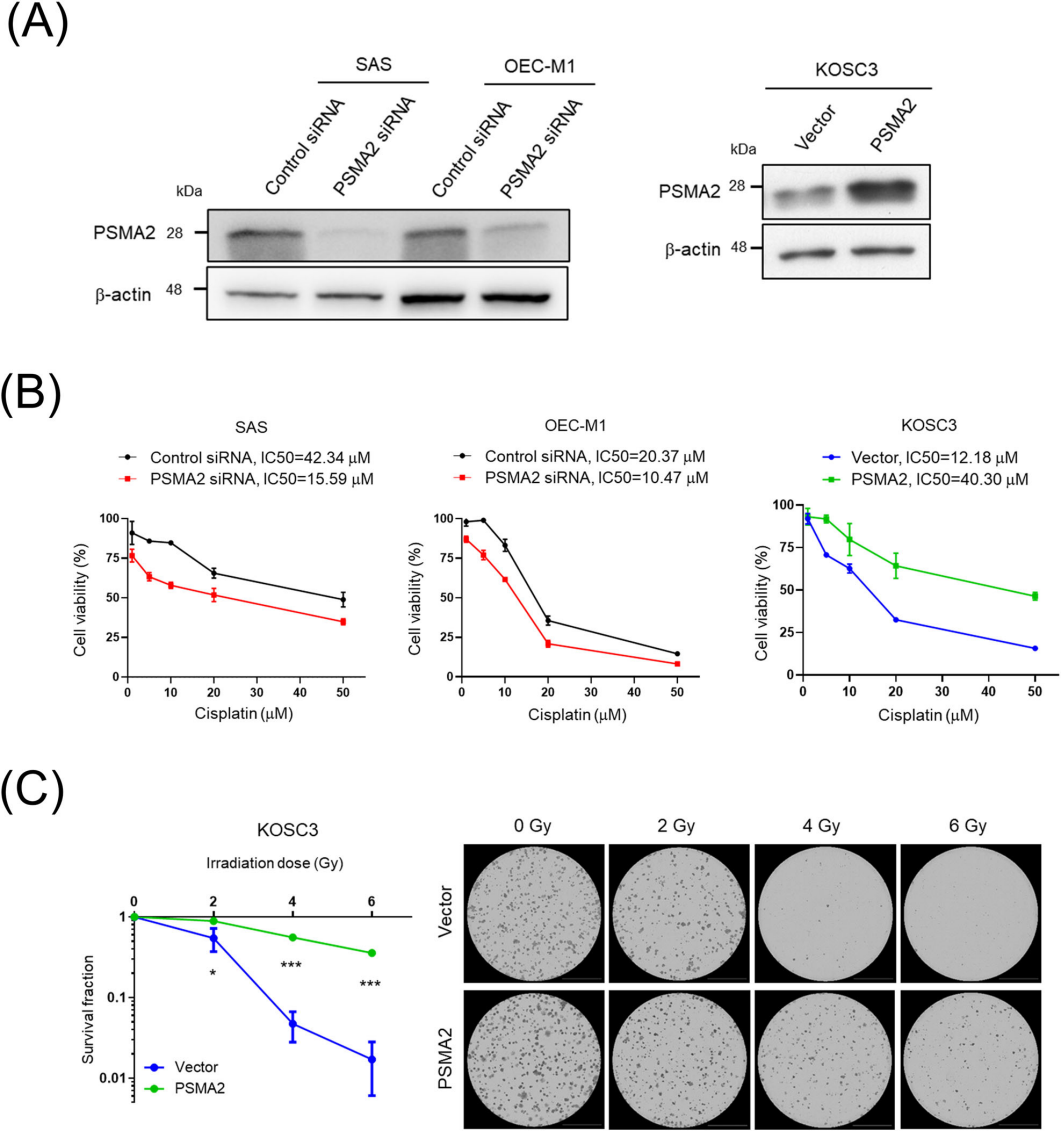
Overexpression of PSMA2 promotes chemo- and radioresistance in OSCC. **A** The efficiency of PSMA2 knockdown and overexpression in OSCC cells determined via Western blot. **B** Chemosensitivities of PSMA2-knockdown and overexpressing OSCC cells were determined based on the relative number of surviving cells after treatment with different doses of cisplatin. Cell survival was examined using the CCK8 assay. **C** Clonogenic survival assays performed with PSMA2-overexpressing and vector cells after exposure to different doses of irradiation.

To further evaluate chemo- and radioresistant role of PSMA2, it was silenced in SAS and OEC-M1 cells and overexpressed in KOSC3 cells, respectively (Fig. 2A). These PSMA2-modified cells were then subjected to cisplatin treatment and irradiation. The results showed that PSMA2-silenced cells exhibited a significant reduction in cisplatin IC50, from 42.34 to 15.59 µM in SAS cells and from 20.37 to 10.47 µM in OEC-M1 cells, compared to control cells. Conversely, PSMA2-overexpressing cells showed an increase in cisplatin IC50, from 12.18 to 40.30 µM in KOSC3 cells, as compared to vector cells (Fig. 2B). Figure 2C demonstrated a significant increase in colony number for PSMA2-overexpressing cells after exposure to irradiation, compared to vector cells.

These findings indicate that PSMA2 contributes to the chemo- and radioresistance phenotype in OSCC cells.

### PSMA2 is involved in cell cycle and mitochondrial dysfunction based on GSEA and IPA analyses

**Fig. 3:**
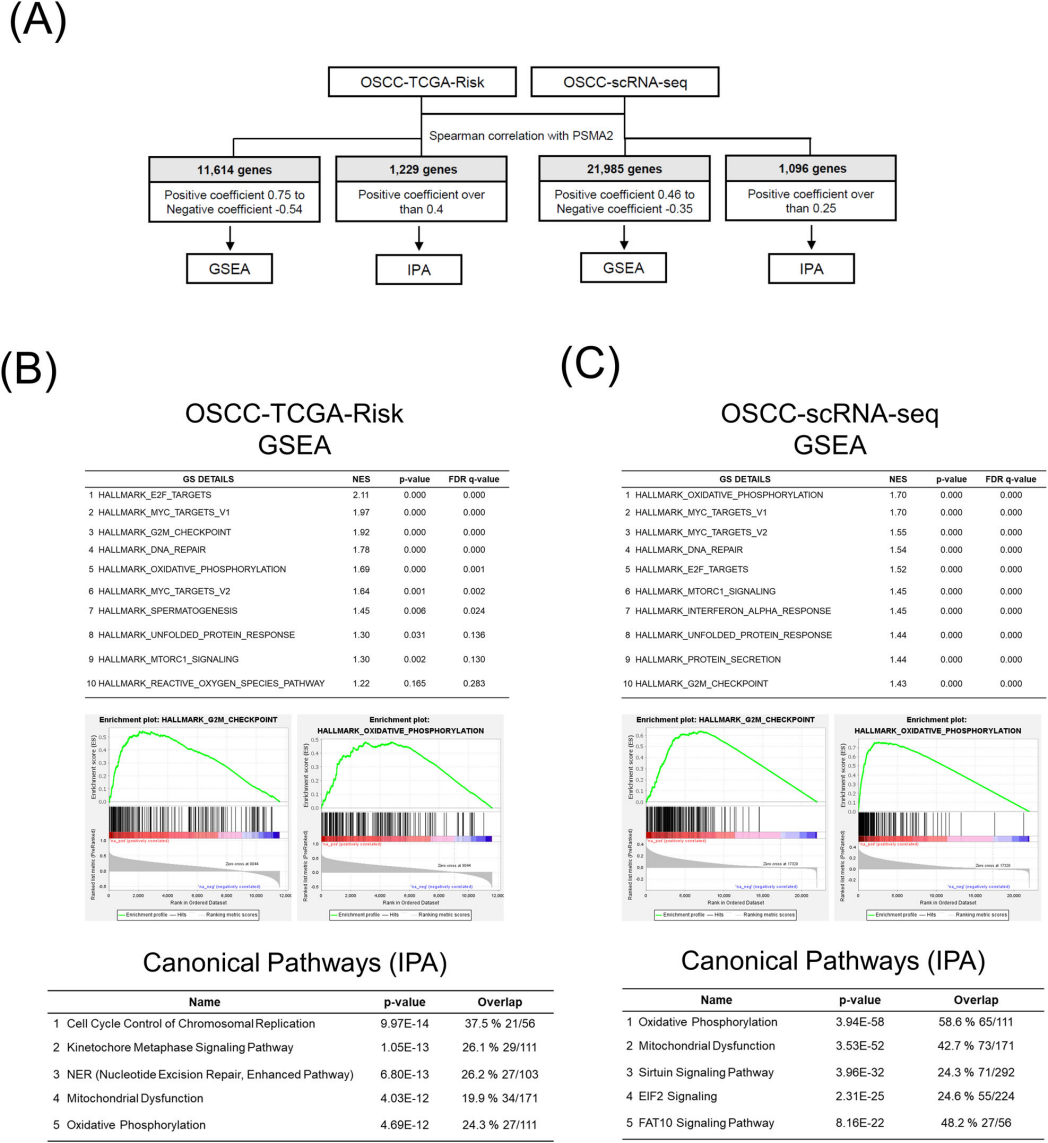
PSMA2 is involved in cell cycle and mitochondrial dysfunction based on GSEA and IPA analyses. **A** Flowchart of GSEA and IPA based on PSMA2-correlated genes. **B**, **C** Enrichment plot and canonical pathways of PSMA2-correlated genes obtained via GSEA and IPA.

To comprehensively profile the molecular pathways associated with PSMA2 in tumorigenesis and chemo-radioresistance in OSCC, we conducted co-expression analysis. Spearman’s correlation coefficient was calculated between PSMA2 and other genes in both OSCC-TCGA-Risk and OSCC-scRNA-seq datasets. The co-expression network and hallmarks of cancer were then analyzed using GSEA. The results were further validated using IPA software (Fig. 3A). Collectively, the findings revealed positive correlations between PSMA2 and signature genes involved in various tumor-promoting functions, particularly those related to cell cycle regulation and mitochondrial dysfunction (Fig. 3B, C). These results suggest that PSMA2 may play a critical role in regulating these pathways in OSCC, potentially contributing to tumorigenesis and chemo- and radioresistance.

### PSMA2 depletion suppresses proliferation and induces G2/M cell cycle arrest and apoptosis via DNA damage signaling

**Fig. 4:**
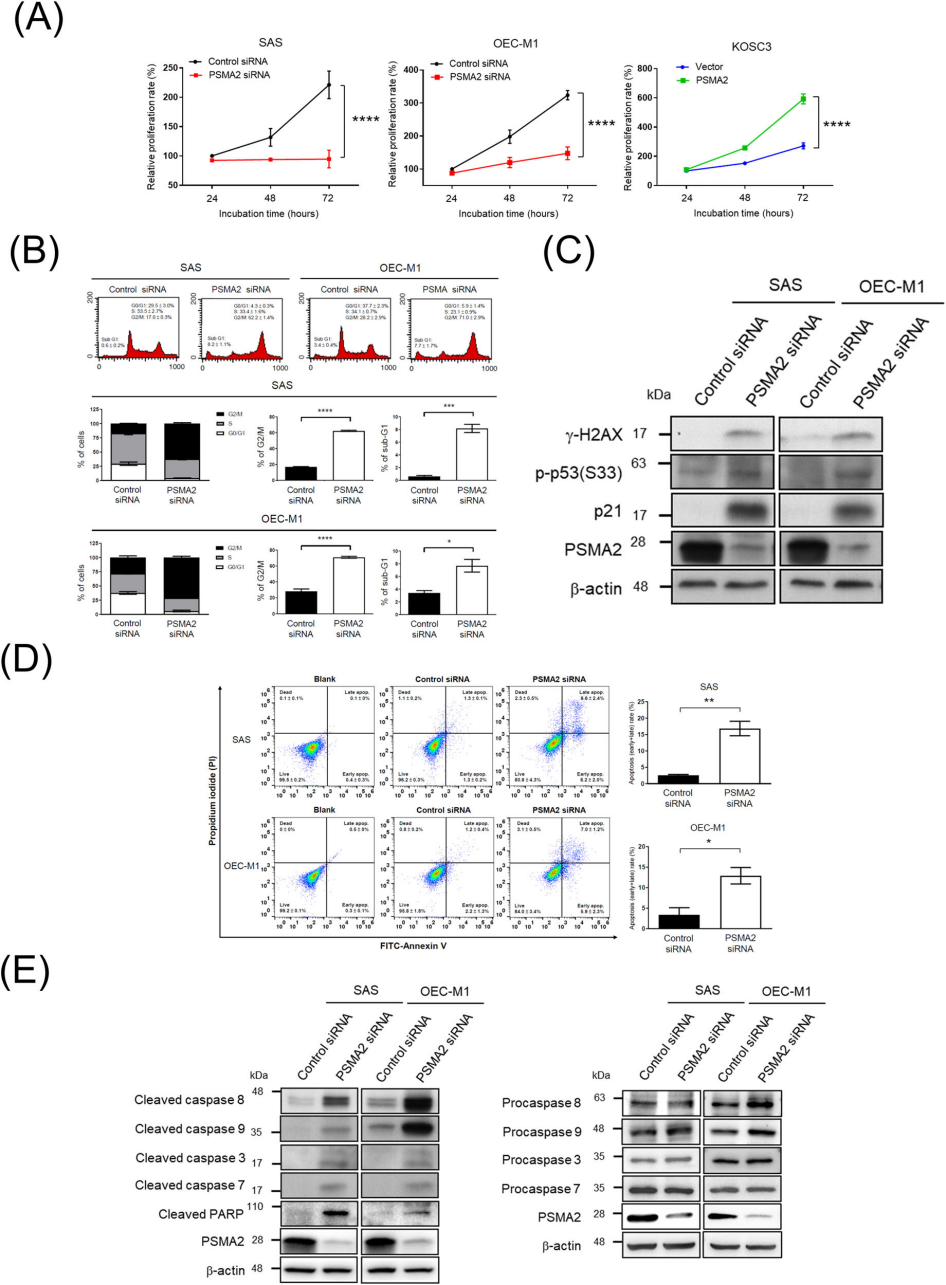
PSMA2 depletion suppresses proliferation and induces G2/M cell cycle arrest and apoptosis via DNA damage signaling. **A** Measurement of cell proliferation curves with the CCK8 assay. **B** Cell cycle was determined by propidium iodide staining followed by flow cytometry analysis. **C** Total cellular proteins of the transfected cells were subjected to Western blotting and analyzed using the indicated antibodies. **D** Apoptosis was determined by annexin V-FITC/PI staining followed by flow cytometry analysis. **E** Total cellular proteins of the transfected cells were subjected to Western blotting and analyzed using the indicated antibodies.

According to the GSEA and IPA pathway analyses, we observed that PSMA2-associated molecules were highly involved in cell cycle pathways. On the basis of this concept, we examined the proliferation and cell cycle distribution in PSMA2-silenced and overexpressing cells. Figure 4A demonstrated that cell proliferation was decreased significantly in PSMA2-knockdown SAS and OEC-M1 cells and elevated remarkably in PSMA2-overexpressing KOSC3 cells. As compared with control siRNA-transfected cells, the percentages of cells in the G2/M phase significantly increased by 45.2% and 42.8% in PSMA2-knockdown SAS and OEC-M1 cells, respectively (Fig. 4B). To confirm these findings, we further determined changes in phosphorylation of H2AX (γ-H2AX), as a sensor of double-strand breaks and its downstream molecule, phosphorylated p53 and p21, which are involved in the G2/M checkpoint pathway. As shown in Fig. 4C, the levels of γ-H2AX and p53 phosphorylation (Ser33) and p21 were remarkably increased in PSMA2-knockdown SAS and OEC-M1 cells as compared with those in the control cells. Because the knockdown of PSMA2 sensitizes cell to DNA damage, followed by G2/M arrest in OSCC cells, the proportion of sub-G1 cells, which is an indicator of apoptosis, also increased in PSMA2-knockdown cells (Fig. 4B). We applied annexin V/PI flow cytometry assays to assess the apoptosis. Figure 4D revealed that the proportions of apoptotic cells significantly increased by 14.26% and 9.52% in PSMA2-knockdown SAS and OEC-M1 cells, respectively. To further confirm these findings, we determined changes in cleavage of caspases and PARP. As shown in Fig. 4E, the cleaved levels of caspase 8, 9, 3, 7, and PARP were remarkably increased, while the levels of procaspases 8, 9, 3, and 7 remained similar between control and PSMA2-knockdown cells.

### PSMA2 overexpression inhibits mitochondrial superoxide production and mitophagy

**Fig. 5:**
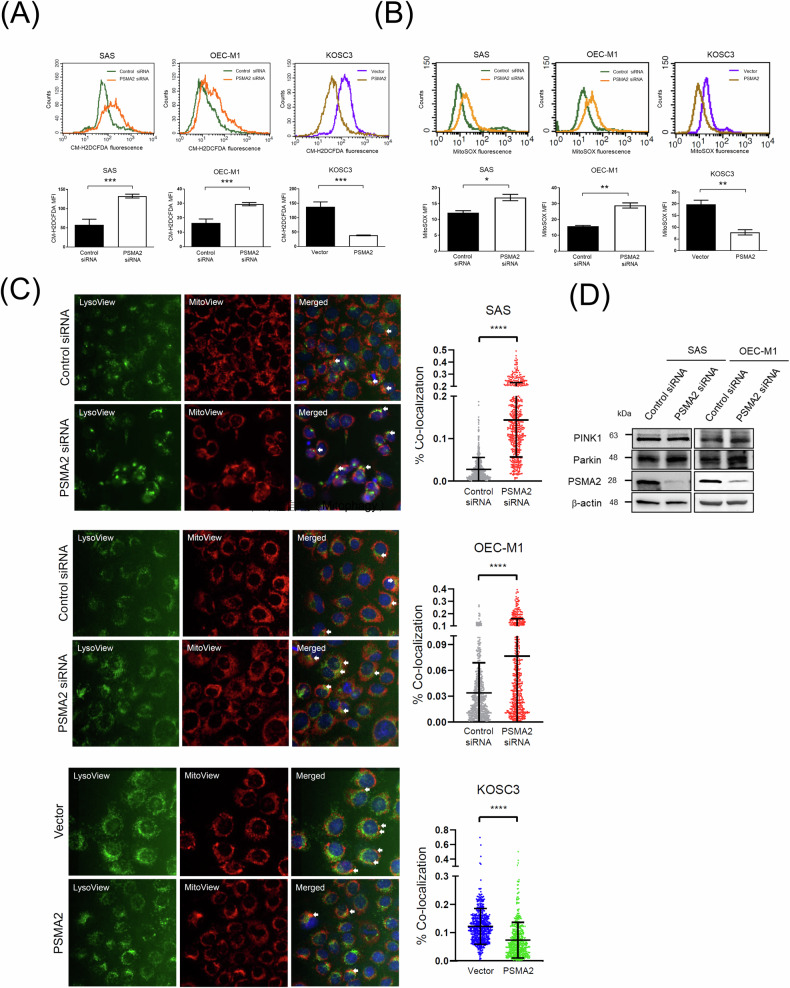
Overexpression of PSMA2 suppresses mitochondrial superoxide production and mitophagy. **A** Cells were stained with CM-H2DCFDA for 30 min followed by flow cytometry for determining the ROS production. **B** Cells were stained with MitoSOX followed by flow cytometry for determining the mitochondrial ROS production. **C** Cells were stained with LysoView (green), MitoView (red) and hoechst 33342 (blue) followed by quantification of MitoView and LysoView co-localization using the INCell analyzer 1000. Arrows indicate co-localized puncta. **D** Total cellular proteins of the transfected cells were subjected to Western blotting and analyzed using the indicated antibodies.

Based to the GSEA and IPA pathway analyses, we found that PSMA2-associated molecules were highly involved in mitochondrial dysfunction pathways. In addition, Fig. 4C demonstrated PSMA2 depletion-induced DNA damage. Therefore, we examined the total ROS and mitochondrial ROS (mtROS) production in PSMA2-silenced and overexpressing cells. As shown in Fig. 5A, B, the total ROS and mtROS production were elevated in PSMA2-knockdown SAS and OEC-M1 cells, and suppressed in PSMA2-overexpressing KOSC3 cells. Previous studies have indicated mtROS are elevated as a consequence of mitophagy induction in some circumstances [19] and mitophagy plays a vital role in cancer progression and chemo- and radioresistance [20–22]. Consistently, Fig. 5C demonstrated the co-localization of lysosome and mitochondrial were significantly suppressed in PSMA2-overexpressing KOSC3 cells and increased in PSMA2-silenced SAS and OEC-M1 cells. Furthermore, we investigated whether PSMA2 regulates mitophagy through PINK1/Parkin signaling. As shown in Fig. 5D, the levels of PINK1 and Parkin are similar in both control and PSMA2-knockdown cells, suggesting that PSMA2 modulates mitophagy via PINK1/Parkin-independent pathway. Taken together, these results indicated that PSMA2 affected the chemo- and radiosensitive phenotype via modulating mitophagy, and it is worth exploring the detail molecular mechanism in the future.

### Mitophagy inducer exhibit antitumor effects in PSMA2-overexpressing OSCC xenograft mouse model

**Fig. 6:**
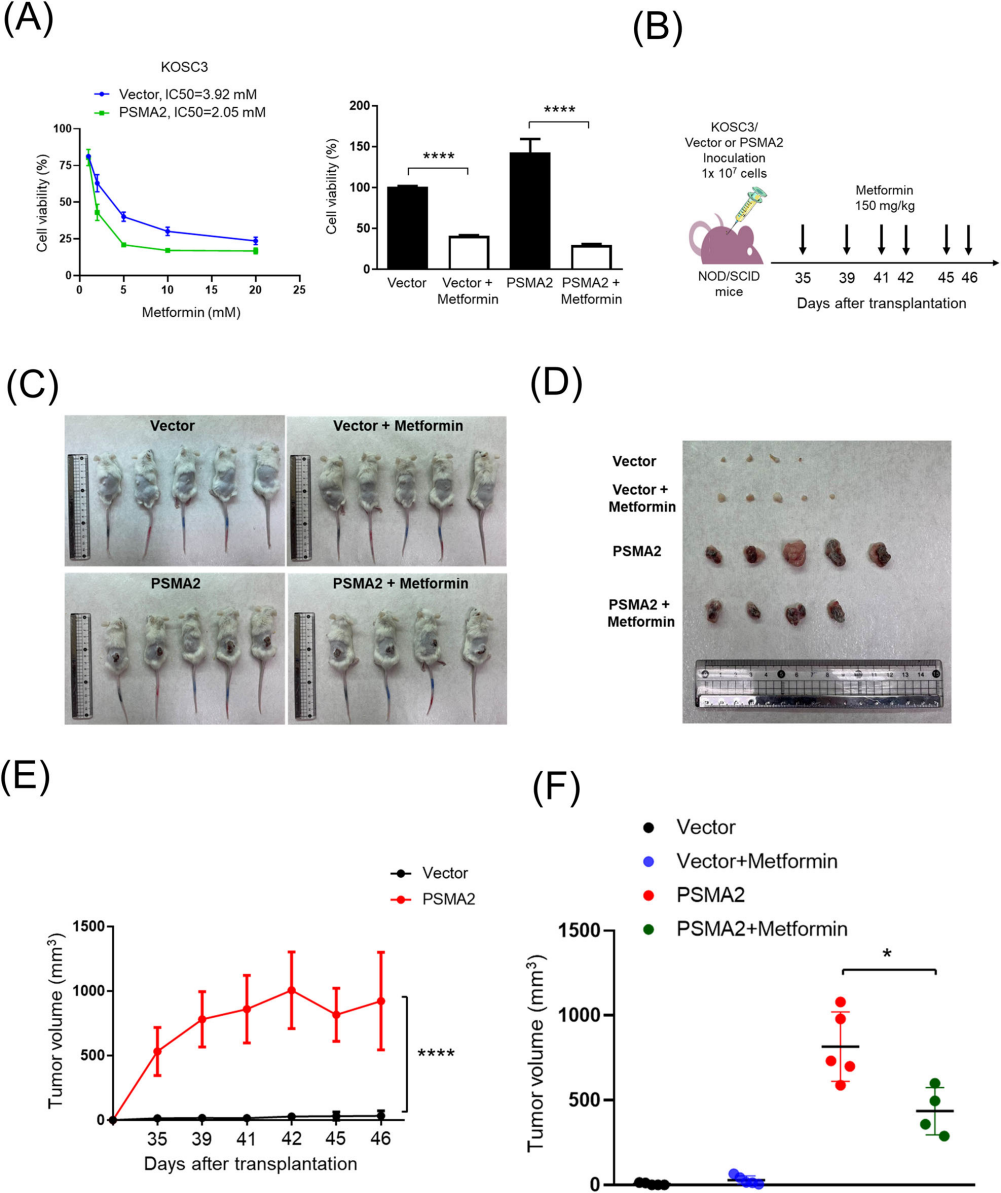
Mitophagy inducer exhibits antitumor effects in PSMA2-overexpressing OSCC xenograft mouse model. **A** Chemosensitivities of vector and PSMA2-overexpressing OSCC cells were determined based on the relative number of surviving cells after treatment with different doses of metformin. Cell survival was examined using the CCK8 assay (left). Quantification analysis of cell viability was performed after metformin (5 mM) treatment for 72 h (right). **B** Experimental schema showing the OSCC xenograft experiments in NOD/SCID mice. **C**, **D** Mice were injected subcutaneously with vector or PSMA2-overexpressing cells, followed by metformin treatment at the indicated time points to observe tumor growth. After sacrifice, tumors from these groups of mice (*n* = 5 for each group; one mouse was missing in the PSMA2-overexpressing group due to unexpected death during the experimental period) were collected. **E** The tumor growth curves are shown. **F** Quantification analysis of tumor volume acquired at the time of sacrifice.

**Fig. 7:**
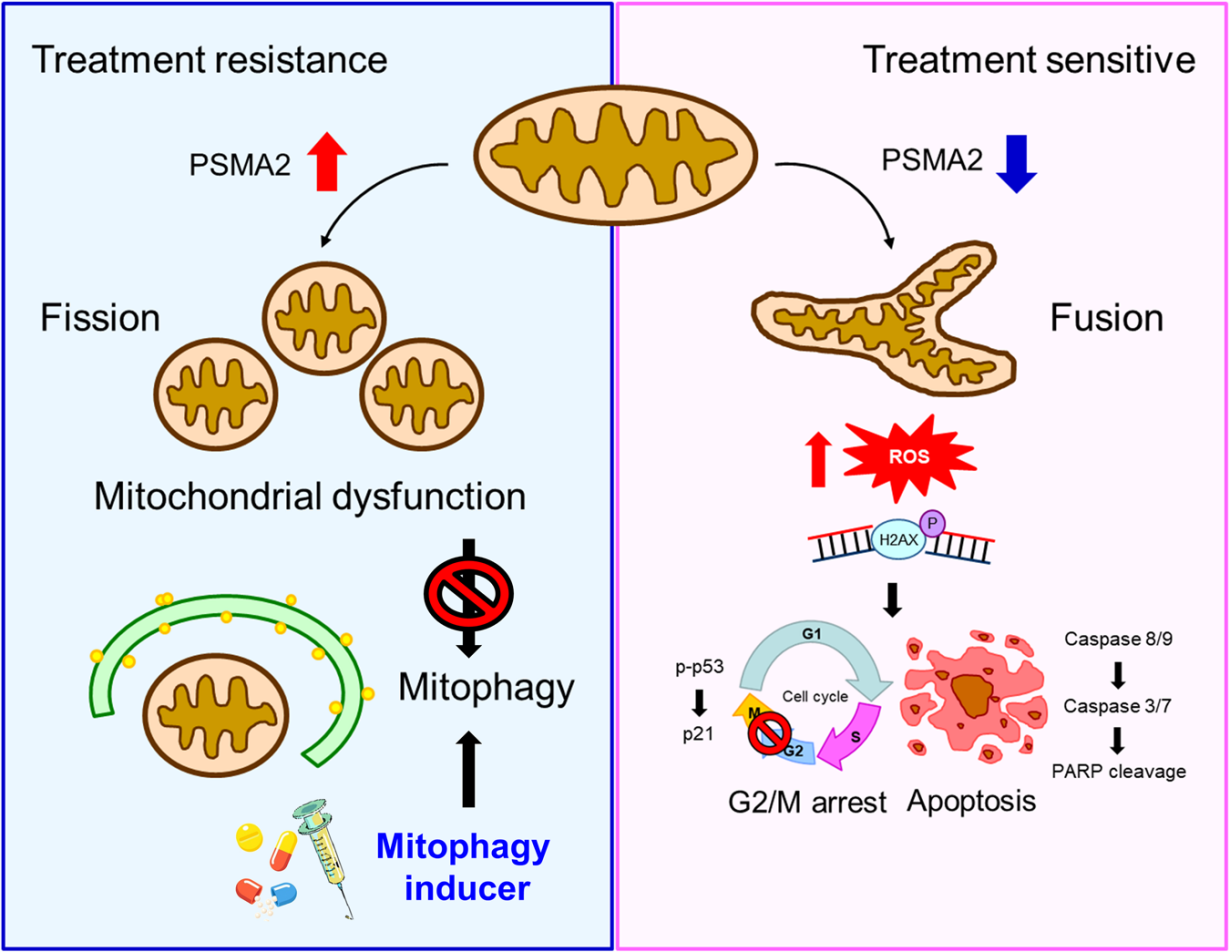
Hypothetical schematic of the resistant role of PSMA2 in OSCC malignant progression. In the OSCC cells, the elevated PSMA2 level triggered mitochondrial dysfunction, which in turn suppressed the ability of mitophagy, contributing to treatment resistance. In contrast, treatment of mitophagy inducer exhibits antitumor effects in PSMA2-overexpressing OSCC. Under the repression of PSMA2 condition, the decreased PSMA2 level induced the levels of ROS, followed by an elevation of G2/M arrest, leading to caspase-dependent apoptosis, as a consequence of inhibition for OSCC progression.

To evaluate the role of the PSMA2-mediated mitophagy pathway in therapeutic efficacy in vitro and in vivo, we used a mitophagy inducer, metformin, for further examination. The results showed that PSMA2-overexpressing KOSC3 cells exhibited a reduction in metformin IC50, from 3.92 to 2.05 mM, compared to control cells, and the treatment significantly suppressed PSMA2-induced tumor growth (Fig. 6A). Next, we established OSCC xenograft mouse models with vector or PSMA2-overexpressing KOSC3 cells. Mice were treated with metformin (150 mg/kg) for 12 days, starting on day 35 post-transplantation (Fig. 6B). No weight loss or severe toxicity was observed in any group during the treatment period (Fig. 6C). As shown in Fig. 6D, E, the overexpression of PSMA2 significantly augmented tumor growth of KOSC3 xenografts in vivo, supporting the oncogenic property of PSMA2 in OSCC tumorigenicity. When compared to untreated controls, metformin treatment demonstrated significant decreases in tumor growth (Fig. 6D, F). Collectively, these results indicate that the mitophagy inducer has stronger antitumor effects in PSMA2-overexpressing OSCC cells (Fig. 7).

## Discussion

In the current study, we propose an effective approach combining TCGA large data analysis and scRNA-seq to unravel the intricacies of oral cancer recurrence and pinpoint key biomarkers. According to the literature search, five have been previously reported as dysregulated proteins or genes in HNSCC, two have been reported as involved in HNSCC recurrence (Table 1). Liu et al. demonstrated that SOD2 promotes the migration and invasion of tongue squamous cell carcinoma [23]. Giotakis et al. showed that cytochrome c immunostaining was observed in 63.3% of patients with malignant parotid tumors [24]. Mesquita et al. indicated that immunoexpression of LGALS7B was observed in 96.9% of OSCC cases and was significantly associated with histological malignancy grading systems [25]. Huang et al. found that PPIA expression in OSCC was significantly higher when compared to normal oral mucosa by immunohistochemistry [26]. Guo et al. predicted a model of six mRNAs, including PCMT1, that could predict HNSCC patient prognosis [27]. In addition, Noh et al. identified and validated a robust gene signature of SOD2-associated genes in HNSCC and confirmed their link to radioresistance [28]. Matsukawa et al. identified LGALS7B as a potential predictive marker of chemotherapy and/or radiotherapy resistance in OSCC by quantitative proteomics and validated it by immunohistochemistry [29]. These results validate the practical feasibility of our proposed method for pinpointing candidate recurrence-associated genes in cancers. Additionally, several studies have demonstrated that integrating single-cell and bulk transcriptome analyses is a powerful strategy for distinguishing cancer subtypes with distinct prognoses and identifying potential cancer biomarkers [30–33]. These findings highlight the molecular heterogeneity of cancer and the advantages of single-cell RNA sequencing in identifying cellular diversities within complex cancer ecosystems. This approach could facilitate accurate patient stratification and the development of individualized, precise treatments.

The core molecular pathways and gene ontology analysis revealed that PSMA2 and its associated signature genes are involved in several critical functions regulating OSCC tumorigenesis and recurrence, such as cell cycle regulation, mitochondrial dysfunction, and the unfolded protein response (UPR) (Fig. 3). Importantly, similar results were observed in previous studies. Rashid et al. used an aptamer-based multiplexed technique to measure human proteins and determine the impact of PSMA2 knockdown on human lung cancer cells [16]. They found that PSMA2 knockdown resulted in significant dysregulation of 52 cellular proteins involved in different biological functions, including the cell cycle, cell death and survival, and cellular stress, including the UPR. Furthermore, we have confirmed the ER stress-related markers, which induce UPR, in our RNA-seq dataset, comparing PSMA2-overexpressing cells to control cells. We found that the level of Activating Transcription Factor 6 (ATF6), a key protein regulated by ER stress, is increased in PSMA2-overexpressing cells. Collectively, we believe that PSMA2 overexpression may influence ER stress and UPR.

Interestingly, accumulating evidence has revealed that PSMA2 is involved in the immune system and signal transduction. Wang discovered that PSMA2 is related to tumor prognosis in acute myeloid leukemia through weighted gene co-expression network analysis. This study builds a risk rating model revealed that PSMA2 is an independent prognostic factor and connected to the immune response [17]. Qi et al. analyzed tumor-associated macrophage-related genes and found that PSMA2, one target of miR-32, is a key regulator in colorectal cancer [14]. Zhu et al. developed a stratification system based on two immune-related ovarian cancer-derived exosome signatures, identifying PSMA2 as a risk factor in immunotherapy treatment for ovarian cancer patients [18]. Collectively, it is worth investigating whether PSMA2 plays a vital role in the immune response in OSCC in the future.

Mitophagy, an important mitochondrial quality control system, selectively degrades excessive or damaged mitochondria through autophagy. Initially, mitophagy was considered an oncosuppressor that maintains cellular homeostasis and prevents oncogenic transformation. Conversely, mitophagy promotes cancer cell survival under cytotoxic stress by degrading damaged mitochondria and reducing mitochondrial ROS [20, 34, 35]. As such, mitophagy acts as a double-edged sword in cancer cell biology, capable of promoting both cancer cell death and survival. There is no consensus on whether mitophagy serves as a suppressor or inducer of cancer drug resistance in specific cancer types, suggesting that the most appropriate treatment should be selected based on the cancer type and anticancer drug. Therefore, mitophagy likely plays a dual role in cancer drug resistance depending on varying conditions and cell types. Herein, we revealed that PSMA2 influences the chemo- and radiosensitive phenotype by modulating mitophagy. Notably, the mitophagy inducer exhibited stronger antitumor effects in PSMA2-overexpressing OSCC cells both in vitro and in vivo (Figs. 5 and 6). We hypothesize that altered expression of PSMA2 provides a direct link between mitochondrial dysfunction and OSCC tumor progression and recurrence, due to defects in mitophagy and fail to degrade excessive or damaged mitochondria. However, there are also potential risks associated with excessive or misregulated mitophagy. For instance, enhanced mitophagy might support tumor survival and proliferation by removing dysfunctional mitochondria, allowing cancer cells to adapt to stressful environments and resist apoptosis [36]. In oral cancer specifically, studies suggest that mitophagy regulated by mitochondrial fission proteins like MTP18 plays a key role in cancer cell survival. Inhibiting mitophagy can lead to increased apoptosis in these cells, suggesting that too much mitophagy may help tumors evade programmed cell death [37]. Furthermore, targeting mitophagy in treatment can also impact normal cells. Excessive mitophagy in healthy tissues may cause unintended mitochondrial damage, reducing energy production and triggering cell death in non-cancerous cells [38]. Therefore, while modulating mitophagy presents a potential therapeutic avenue, careful balance is required to avoid enhancing cancer cell survival or harming normal tissues. Further studies are warranted to elucidate how mitophagy is regulated in different cancer types and during anticancer drug treatments, which may enlighten the development of novel strategies for cancer treatment. Collectively, this study provides a molecular and biochemical rationale for developing therapeutic strategies to selectively kill PSMA2-overexpressing OSCC cells using mitophagy inducer compounds.

## Materials and methods

### Patient populations and clinical specimens

A total of 86 patients whose untreated OSCC tumors were primarily managed by surgical resection with subsequent CCRT at the Chang Gung Memorial Hospital (CGMH) from 2011 to 2019 were enrolled following informed consent.

### Ethical statement

This study was approved by the Institutional Review Board of CGMH (No. 202001759B0 of IRB). Written and informed consent was obtained from all participants. We confirmed that all methods were performed in accordance with relevant guidelines and regulations.

### Cell culture

SAS cells (Prof Chien, Chang Gung University) were grown in Dulbecco’s modified Eagle’s medium (DMEM) (Invitrogen, Carlsbad, CA) containing 10% fetal bovine serum (FBS) (Gibco BRL, Carlsbad, MD) plus antibiotics. KOSC3 cells (Dr Chu, Chang Gung University) and OEC-M1 cells (Prof Tsai, Chang Gung University) were cultured in Roswell Park Memorial Institute medium 1640 (Invitrogen) containing 10% FBS plus antibiotics. The cells were cultured at 37 °C in a humidified atmosphere of 95% air and 5% CO_2_. Cell cultures were determined to be Mycoplasma free by DNA fluorochrome staining with Hoechst 33258 bisbenzimide. Short tandem repeat (STR) profiling was utilized to authenticate all the cell lines.

### Antibodies

All commercial antibodies used in this study are listed as follows: PSMA2 antibodies (sc-377148, Santa Cruz Biotechnology, Inc., USA), β-actin antibodies (MAB1501, Sigma-Aldrich, MO, USA), γ-H2AX antibodies (AP0099, ABclonal Technology, USA), p-p53 (Ser33) antibodies (AP0762, ABclonal Technology), cleaved caspase eight antibodies (#9496, Cell Signaling, MA, USA), cleaved caspase nine antibodies (#9915, Cell Signaling), cleaved caspase three antibodies (#9915, Cell Signaling), cleaved caspase seven antibodies (#9915, Cell Signaling), cleaved PARP antibodies (#9915, Cell Signaling), procaspase eight (#12742, Cell Signaling), procaspase nine antibodies (#12742, Cell Signaling), procaspase three antibodies (#12742, Cell Signaling), procaspase seven antibodies (#12742, Cell Signaling), PINK1 antibodies (A7137, ABclonal Technology), Parkin antibodies (A11172, ABclonal Technology).

### Gene knockdown of PSMA2 using small interfering RNA

OSCC cells were transfected with the Dharmacon ON-TARGETplus Nontargeting Control Pool (Thermo Fisher Scientific, Rockford, IL) and targeting genes, which contained a pool of four siRNAs by Lipofectamine RNAiMAX reagents (Invitrogen, CA, USA). The siRNA sequences are listed as follows: PSMA2 (GCAUAUAGGUUUGGUGUAC, ACACAAAGUAGAACCAAUU, GAAUGAGGGACGACCAUAU, CAAAUGGUGUGGUAUUAGC).

### Cell cycle and apoptosis assay

For cell cycle analysis, cells were harvested by trypsinization and fixed in 70% ice-cold ethanol comprising 2 mg/mL RNase for 30 min and were finally stained with propidium iodide (PI; 50 mg/mL) for 10 min. The fluorescence of PI in transfected cells (1 × 10^4^) was determined via flow cytometry analysis (FACScan System, Becton Dickinson, San Diego, CA) and counted the percentage of cells using CellQuest programs. For apoptosis detection, a commercial kit containing annexin V-FITC and PI was used (Strong Biotech Corporation, Taiwan). The cells (1 × 10^4^) were analyzed using a FACSCalibur flow cytometer (BD Bioscience, San Jose, CA) after annexin V-FITC and PI staining.

### Clonogenic survival assay

The transfected cells were plated into 6-well plates. After culture for 24 h, the cells were subjected to irradiation (0, 2, 4, or 6 Gy). For colony formation, the irradiated cells were cultured for additional 7 days. The colonies were fixed with acetic acid/methanol solution at a 1:3 ratio and then stained with 0.5% crystal violet solution. The number of colonies (>50 cells/colony) were counted and analyzed by Lionheart FX automated microscope (BioTek, Vermont, USA). The survival fraction of each irradiation group was corrected by the non-irradiated groups. Dose‑response clonogenic survival curves were plotted on a log‑linear scale. The average data were fitted to a multi-target single‑hit model.

### RNA extraction and quantitative real-time polymerase chain reaction (qRT-PCR)

Total RNA was extracted and converted into cDNA from OSCC tumor and normal counterpart tissues as described previously [39]. qRT-PCR was performed using the SYBR Green system. Fluorescence emitted by SYBR Green was observed using the ABI PRISM 7500 sequence detection system (Applied Biosystems, Werrington, UK). The primer sequences are listed below: PSMA2 (F: 5′-CTGGAGCTTACTTTGCCT GGAAA-3′, R: 5′-CCAGCTTCATTGCAGATTCCAA-3′), GAPDH (F: 5′-CATGTTCCATATGATTCCAC-3′, R: 5′-CCTGGAAGATGGTGATG-3′).

### Cell proliferation assay

After transfection for 24 h, OSCC cells were harvested by trypsinization and suspended at a density of 4 × 10^2^ cells/100 μl in a 96-well plate (100 μl per well). Cell viability was evaluated with Cell Counting Kit-8 (CCK-8) (BIOTOOLS Co., Ltd Taiwan) according to the manufacturer’s protocol. Briefly, cells in each well were incubated with 10 µl CCK‑8 reagent at 37 °C for 2 h. The optical density was measured at a wavelength of 450 nm using an ELISA reader (Molecular Devices, SpectraMax M2).

### Detection of ROS production

The transfected cells were incubated for 30 min with 10 μM CM-H2DCFDA (Thermo Fisher Scientific, MA, USA) at 37 °C. The mean fluorescence intensity was analyzed by flow cytometry based on the manufacturer’s instructions.

### Assessment of the mitophagy

Cells were stained with LysoView™ (green) (Biotium, USA), MitoView™ (red) (Biotium), and Hoechst 33342 (blue) (Biotium) followed by quantification of MitoView and LysoView co-localization using the automated epifluorescence microscopy InCell Analyzer 1000 (GE Healthcare, USA). In brief, the nuclei were identified based on the Hoechst 33342 staining, and cells were defined using a 4- to 8-μm collar. Mitophagy events were determined from the percentage of co-localization between LysoView™ and MitoView™. The percentage of red pixels overlapping with green pixels normalized to total red pixels. Thirty different fields were imaged in each well using 40× magnification, which covered around 500–800 cells per well. All images were quantified using a Multi-Target Analysis module of the InCell Investigator software (GE Healthcare).

### Xenograft tumor formation

For the tumor propagation assay, vector control or PSMA2-overexpressing KOSC3 cells were mixed with Matrigel and subcutaneously injected into 6-week-old male NOD SCID mice (1 × 10^7^ cells each). Five weeks later, the mice were randomly divided into two groups and administered Metformin (150 mg/kg) intraperitoneally once every 2 days. Two weeks after the drug injection, the mice were sacrificed, and the tumors were harvested and weighed. Tumor volume was calculated using the following equation: length × width^2^. The experiments were conducted in a randomized and observer-blinded manner. All animal experiments were performed in accordance with the National Cheng Kung University Institutional Animal Care and Use Committee Guide for the Care and Use of Laboratory Animals.

### Statistical analysis

The Wilcoxon test was used to analyze the qPCR results from OSCC and normal counterpart tissues. The Cox regression, hazard ratio estimation, and 95% confidence intervals for hazard ratios were calculated using the R package survival analysis. A comparison of survival rates was carried out with the log-rank test, and the results were visualized with Kaplan–Meier plots. Statistical analyses were performed using GraphPad Prism V5.01 (GraphPad Software, Inc., California, USA). Student’s *t*-test was used to compare two groups. Two-tailed *p*-values of 0.05 or less were considered statistically significant.

## Supplementary information

Supplemental Material-raw blot-R

## Data Availability

The data supporting the findings of this study are available within the article.
