# Supplementary figures and images for: PSMA2 promotes chemo- and radioresistance of oral squamous cell carcinoma by modulating mitophagy pathway
Source: Cell Death Discov. 2025 Jan 10;11:2. doi: 10.1038/s41420-025-02286-2 (PMC11724067; doi:10.1038/s41420-025-02286-2)

Fig. 2A

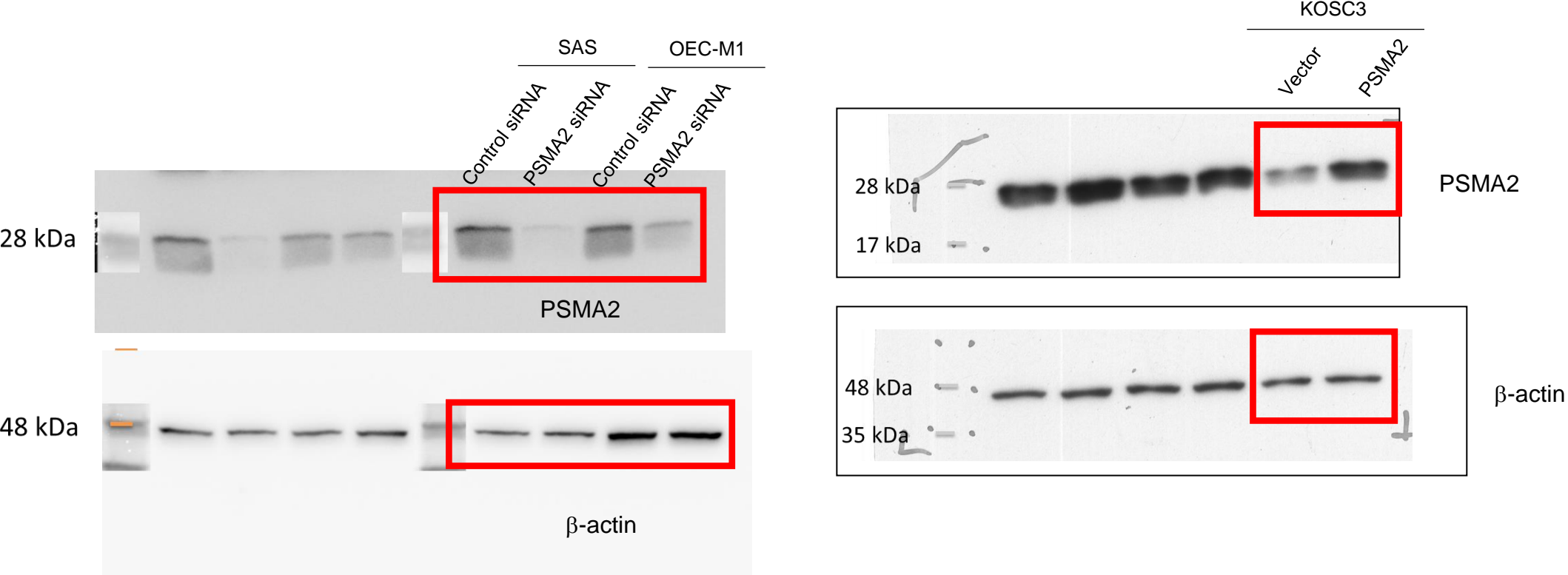

Fig. 4C

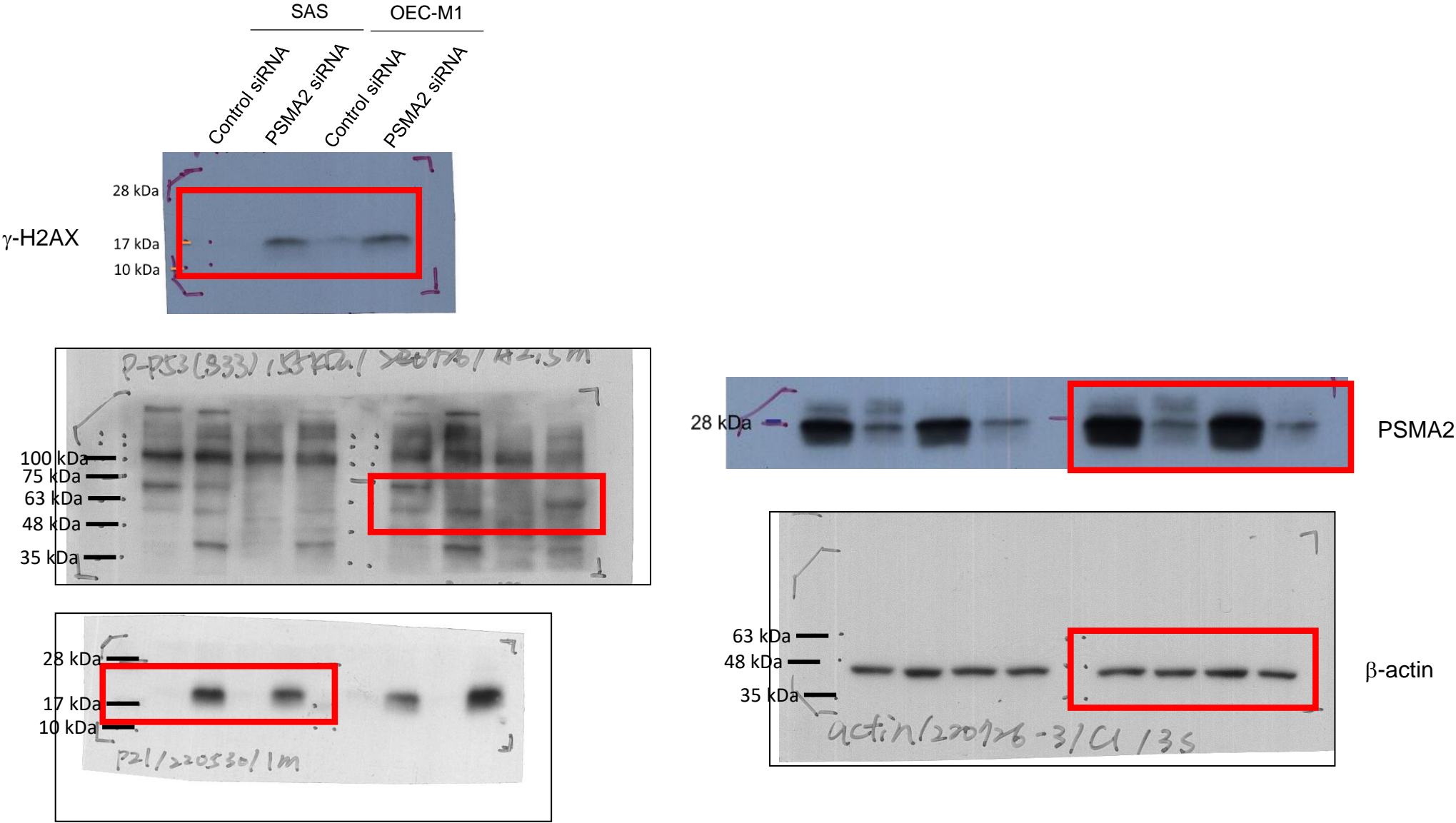

Fig. 4E

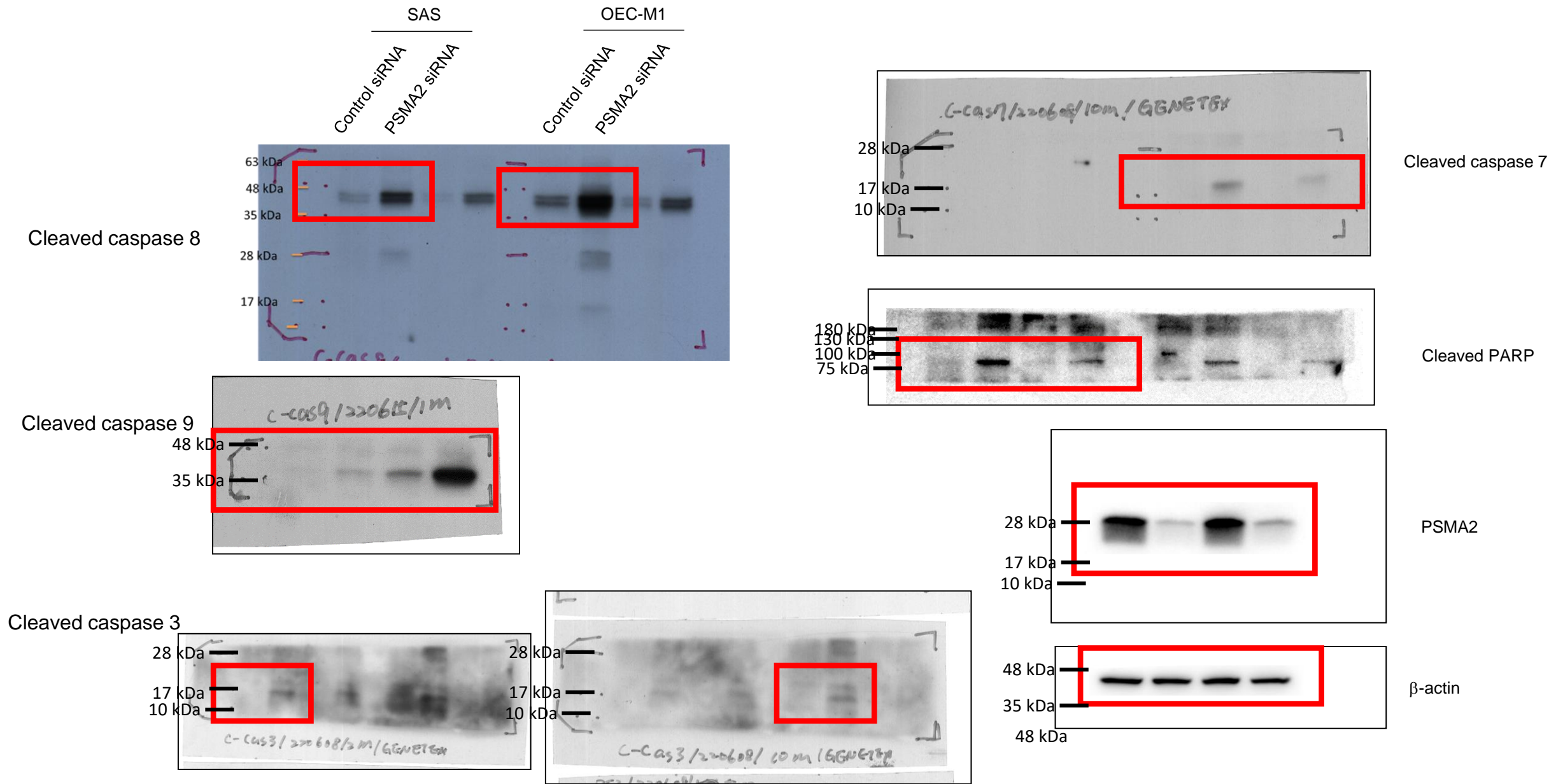

Fig. 4E

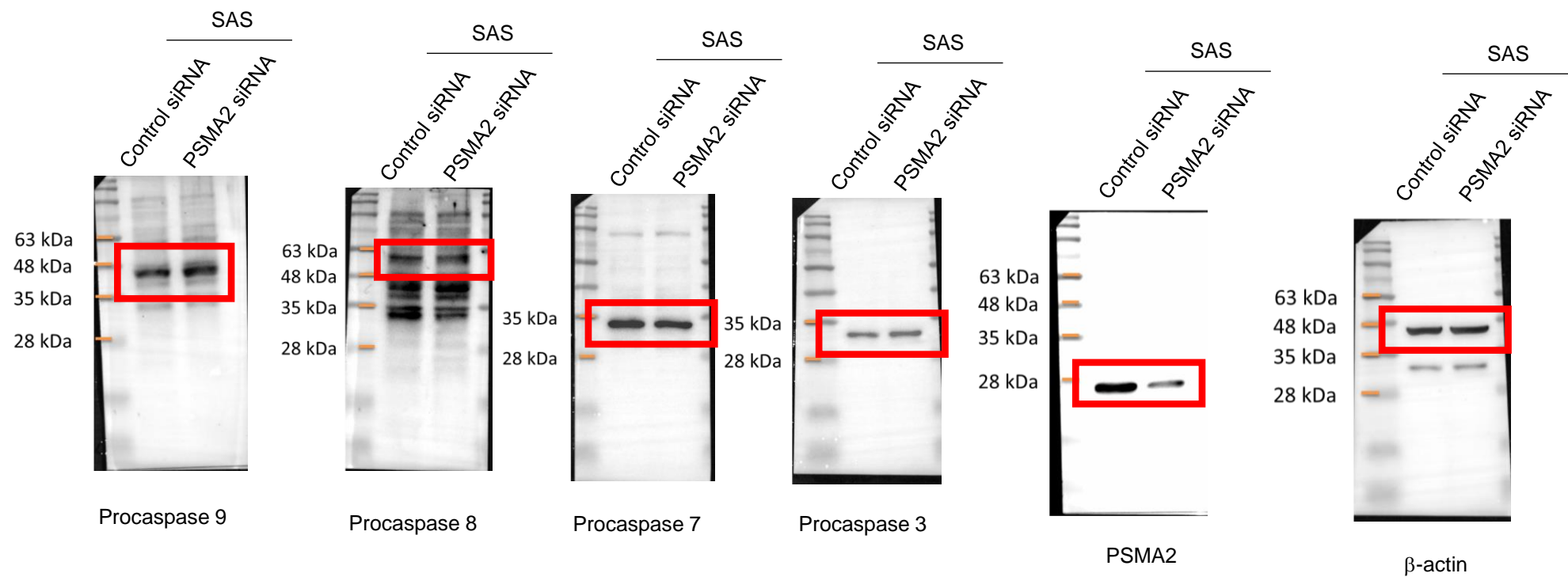

Fig. 4E

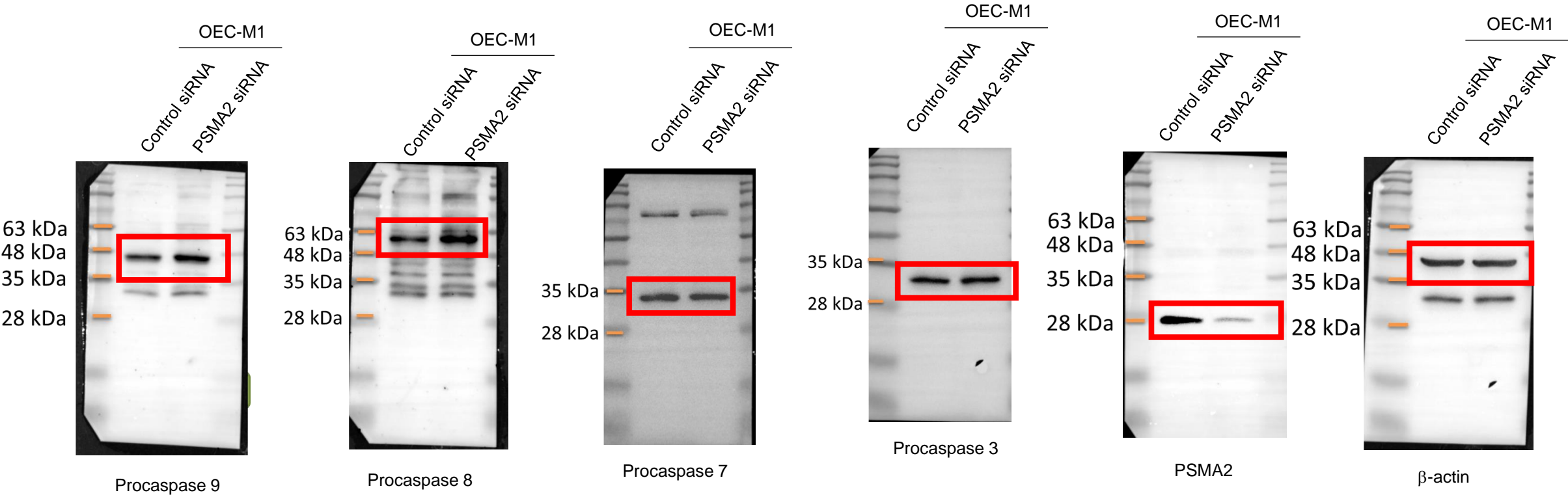

Fig. 5D

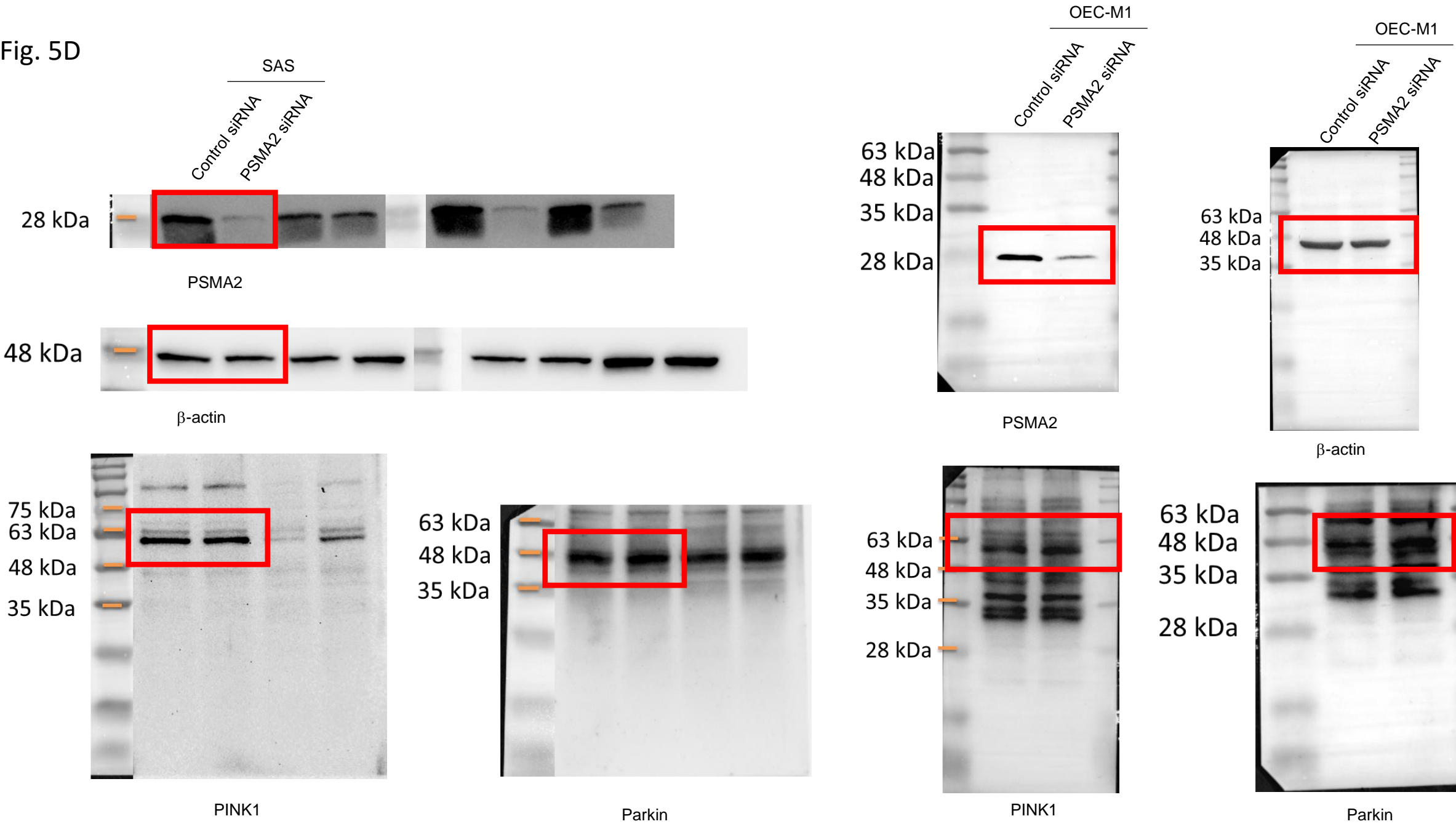

Supplement: Supplementary file 1 — Supplemental Material-raw blot-R [file 41420_2025_2286_MOESM1_ESM.pdf]
